# Complete Genome Sequences of Four Strains from the 2015-2016 *Elizabethkingia anophelis* Outbreak

**DOI:** 10.1128/genomeA.00563-16

**Published:** 2016-06-16

**Authors:** Ainsley C. Nicholson, Anne M. Whitney, Brian D. Emery, Melissa E. Bell, Jarrett T. Gartin, Ben W. Humrighouse, Vladimir N. Loparev, Dhwani Batra, Mili Sheth, Lori A. Rowe, Phalasy Juieng, Kristen Knipe, Christopher Gulvik, John R. McQuiston

**Affiliations:** a Special Bacteriology Reference Laboratory, Bacterial Special Pathogens Branch, Division of High Consequence Pathogens and Pathology, Centers for Disease Control and Prevention, Atlanta, Georgia, USA; b Division of Scientific Resources, Centers for Disease Control and Prevention, Atlanta, Georgia, USA; c Division of Healthcare Quality and Promotion, Centers for Disease Control and Prevention, Atlanta, Georgia, USA

## Abstract

The complete circularized genome sequences of selected specimens from the largest known *Elizabethkingia anophelis* outbreak to date are described here. Genomic rearrangements observed among the outbreak strains are discussed.

## GENOME ANNOUNCEMENT

In 2016, an outbreak centered in Wisconsin was originally attributed to *Elizabethkingia meningoseptica*. Using matrix-assisted laser desorption ionization–time of flight mass spectrometry (MALDI-TOF MS) with an in-house database, optical mapping of genomic DNA, and whole-genome sequences, we were able to identify the agent as *Elizabethkingia anophelis*. Described in 2011 (1), *E. anophelis* belongs to the historically defined *Elizabethkingia* genomospecies 1 (2). The genomospecies of *Elizabethkingia* display no consistent distinguishing phenotypic characteristics (3); therefore, advanced identification techniques are required to differentiate them.

Isolates were grown on heart infusion agar supplemented with 5% rabbit blood at 35°C. DNA was extracted using the Zymo Fungal/Bacteria DNA MicroPrep kit (Zymo Research Corporation, Irvine, CA). Libraries were prepared using the NEBNext Ultra DNA library prep kit for Illumina (New England BioLabs, Ipswich, MA), and sequence reads were generated using the Illumina MiSeq reagent kit version 2 and MiSeq instrument (Illumina, Inc., San Diego, CA). *De novo* assemblies were prepared using the CLC Genomics Workbench version 8.0.3 assembler (CLC bio, Waltham, MA) using reads that were trimmed for quality (limit, 0.02%). Low-coverage contigs and contigs <500 bp were excluded. Optical mapping with *NcoI* using the OpGen Argus system (Gaithersburg, MD) allowed ordering and orientation of all contigs. Sequence linkages between adjacent contigs were resolved using read alignments.

Nonidentical repetitive regions in the CSID_3015183678 genome were resolved using data generated on the Pacific Biosciences RSII instrument (Pacific Biosciences, Menlo Park, CA). DNA was extracted using the Epicentre MasterPure kit (Madison, WI), and 20-kb SMRTbell libraries were generated using the Pacific Biosciences DNA template preparation kit and sequenced on one SMRTcell using C4 chemistry, with a movie time of 360 min. A *de novo* assembly was conducted using PacBio’s Hierarchical Genome Assembly Process (HGAP3, SMRT Analysis 2.3.0) (4), resulting in a complete closed assembly of a single contig.

Outbreak strain reads were mapped to the strain CSID_3015183678 genome; Table 1 shows accession numbers for strain CSID_3015183678 and three additional outbreak strains that were selected for this publication. Unmapped reads were collected and *de novo* assembled; no insertions or episomal elements were found. Two deletions were found in strain CSID_3000521207. An in-frame 1,515-bp deletion at position 3142444 (positions of the genome features are based on strain CSID_3015183678) joins two adjacent S41 family peptidases into a new hybrid S41 family peptidase. The 76,250-bp deletion at position 3779423 removes 77 protein-coding genes.

**TABLE 1:** BioSample and accession numbers

Strain	BioSample no.	Accession no.	Rearrangement configuration
CSID_3015183678	SAMN04567744	CP014805	A-Brc-Crc
CSID_3015183681	SAMN04567745	CP015068	C-B-Arc
CSID_3015183684	SAMN04590540	CP015066	A-B-Crc
CSID_3000521207	SAMN04567738	CP015067	C-Brc-Arc

Three segments, A, B, and C at position 3929927, appear to undergo ordered rearrangement. B in the middle can be present in either direct or reverse complement (rc) orientation; flanking elements A and C exchange locations and will be in either direct and or reverse complement, depending on location, resulting in four configurations for the region: A-B-Crc, C-B-Arc, A-Brc-Crc, and C-Brc-Arc. Among all of the outbreak strains, isolates have reads consistent with one, two, or more configurations. This indicates that the region can be stable or may undergo rearrangement during cell growth.

The genomes were annotated by the NCBI Prokaryotic Genome Annotation Pipeline. Reads for the strains associated with this outbreak have been deposited in the Sequence Read Archive (SRA).

### Nucleotide sequence accession numbers.

The complete genome sequences have been deposited at GenBank under BioProject no. PRJNA315668. The accession and BioSample numbers for each strain are shown in Table 1.
